# Contribution of Vasoactive Intestinal Peptide to the Depressant Effects of Glucagon-like Peptide-2 on Neurally Induced Contractile Responses in Mouse Ileal Preparations

**DOI:** 10.3390/ijms262411797

**Published:** 2025-12-06

**Authors:** Maria Caterina Baccari, Donata Conti, Maria Giuliana Vannucchi, Eglantina Idrizaj

**Affiliations:** 1 Section of Physiological Sciences, Department of Experimental & Clinical Medicine, University of Florence, 50134 Florence, Italy; mcaterina.baccari@unifi.it; 2 Research Unit of Histology & Embryology, Department of Experimental & Clinical Medicine, University of Florence, 50139 Florence, Italy; donata.conti@unifi.it (D.C.); mariagiuliana.vannucchi@unifi.it (M.G.V.)

**Keywords:** glucagon-like peptide-2 (GLP-2), vasoactive intestinal peptide (VIP), ileal contractile activity, neuromodulation

## Abstract

Glucagon-like peptide-2 (GLP-2) has been reported to cause gastrointestinal relaxation by interfering with enteric inhibitory neurotransmitters, including vasoactive intestinal peptide (VIP). However, the involvement of VIP in the GLP-2’s actions on isolated ileal preparations has never been explored. In this study, we investigated whether VIP contributes to the inhibitory effects of GLP-2 on spontaneous and neurally evoked contractions in mouse ileal segments. Functional experiments showed that VIP, as well as GLP-2, depresses both spontaneous and electrically induced contractile responses. The VIP antagonist, VIP 6–28, slightly increased the amplitude of the neurally induced contractile responses. VIP 6–28 did not alter the hormone’s effects on the spontaneous activity, but reduced its inhibitory action on the neurally evoked contractions. In GLP-2-exposed specimens, immunohistochemistry showed a significant decrease in VIP-positivity in nerve fibers located in the muscle layers. These results provide the first evidence that in isolated mouse ileal preparations VIP contributes to the inhibitory effects of GLP-2 on the neurally induced contractile responses. From a physiological point of view, such depressant effects of the hormone may represent a mechanism aimed at slowing intestinal transit and optimizing nutrient absorption.

## 1. Introduction

Glucagon-like peptide-2 (GLP-2) is a 33-amino acid proglucagon-derived peptide, mainly secreted by enteroendocrine L-cells chiefly located in the distal ileal and colonic mucosa [1,2]. Nutrients are the major stimuli for the hormone secretion into the bloodstream [3,4]. GLP-2 exerts its effects by binding to a G protein-coupled receptor (GLP-2R) which is expressed, not only in the central nervous system [5], but also in the gastrointestinal tract [6]. In the gut, the hormone has been reported to be involved in a variety of functions, in addition to its intestinotrophic effects for which it was primarily known [7]. In both humans and animals, GLP-2 plays a key role in regulating the secretion of various enzymes involved in the digestion of nutrients and in promoting their absorption [8,9,10,11,12,13,14], thereby contributing to metabolic control and maintenance of a positive energy balance [15,16,17].

GLP-2 has also been reported to influence gastrointestinal motility in rodents by acting both centrally and peripherally [18,19,20,21]. Regarding peripheral actions, GLP-2 has been reported to induce gastrointestinal relaxant effects in mammals through a prejunctional modulatory action on neurotransmitter release, consistent with the expression of GLP-2R on both excitatory and inhibitory enteric neurons [21,22]. GLP-2 has been shown to depress spontaneous and electrically evoked contractions. Specifically, the hormone exerts a negative control on cholinergic neurotransmission by decreasing acetylcholine release from enteric neurons in both small and large intestinal preparations from mice [21,23,24]. In addition to decreasing the excitatory input to the smooth muscle, the hormone has also been reported to increase the inhibitory input. In this view, among the various signaling pathways activated by GLP-2R [2,25], nitric oxide (NO) and vasoactive intestinal peptide (VIP) have been identified as key mediators in many of the hormone’s actions within the gastrointestinal tract, including motility [26,27]. NO and VIP are indeed considered as the major non-adrenergic, non-cholinergic (NANC) inhibitory neurotransmitters known to relax the gastrointestinal smooth muscle [28,29]. In particular, GLP-2 has been reported to enhance both NO production [30] and VIP release [31] in mouse gastric strips. An increased VIP release has also been observed in the isolated whole stomach [32] and in duodenal preparations from rodents [21]. Notably, the GLP-2R has been found on endothelial nitric oxide synthase (eNOS)- and VIP-positive enteric neurons [33].

Although GLP-2R expression and GLP-2 production have been revealed in the ileum [34], the effects of the hormone on the motility of this isolated segment and its mechanism of action have been poorly investigated. We recently provided the first evidence [23] that the hormone depresses mouse ileal preparations’ contractility, and this effect appears to occur through a dual opposite modulatory action on both excitatory cholinergic and inhibitory nitrergic neurotransmission. However, even if the recruitment of VIP in the effects of GLP-2 has been documented in various regions of the gastrointestinal tract [21,31,32], the potential involvement of VIP signaling in GLP-2-induced modulation of isolated ileal motor responses remains entirely unexplored. This gap in knowledge is particularly relevant given the well-established importance of ileal motor function in several physiological processes, including nutrient absorption.

Therefore, based on all the above observations, the present study aims to investigate, through a combined functional and immunohistochemical approach, whether VIP signaling participates in the inhibitory actions of GLP-2 on both spontaneous and neurally induced contractions in ileal segments from mice.

## 2. Results

### 2.1. Functional Experiments

#### 2.1.1. Ileal Spontaneous Activity

**Figure 1:**
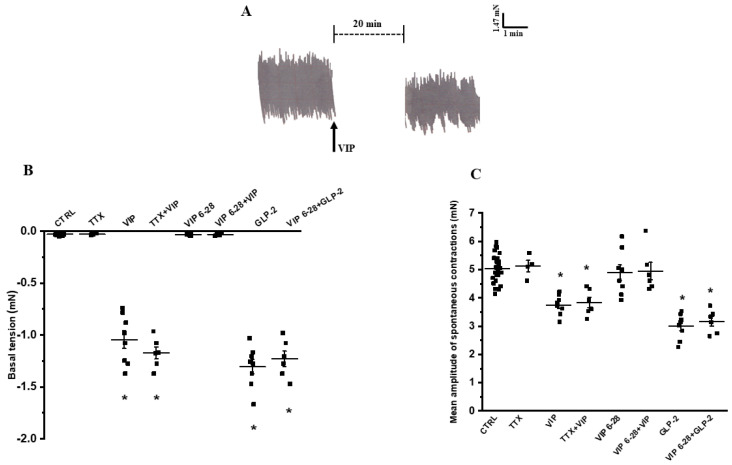
Effect of VIP on basal tone and spontaneous contractions in ileal preparations. (**A**): Representative recording showing spontaneous contractility (left). The addition of VIP (100 nmol/L) to the bath medium slightly reduces basal tension and decreases the amplitude of spontaneous contractions (right). (**B**,**C**): Statistical analysis on the influence of VIP on either basal tension (**B**) or mean amplitude of spontaneous contractions (**C**). Both parameters are not altered by TTX (**B**,**C**), whereas VIP affects them, inducing a modest decay of the basal tension (**B**) and a decrease in amplitude of the spontaneous contractions (**C**) when compared to their respective controls (CTRL). The inhibitory effects of VIP are unaffected by TTX (TTX + VIP). The VIP antagonist, VIP 6–28 (10 µmol/L) does not cause any action per se but prevents the actions of VIP on both basal tension and spontaneous contractions (VIP 6–28 + VIP). GLP-2 causes a decay of the basal tension and a reduction in amplitude of the spontaneous contractions, and these effects were not modified by VIP 6–28 (VIP 6–28 + GLP-2) (**B**,**C**). Note the different *y*-axis scales between the two histograms. Values obtained following the addition of each substance to the organ bath were compared with their respective controls. As no significant differences were found among control values for the two parameters, data were analyzed as a single control (CTRL) group. Values are expressed as mean ± SE (*n* = 4–8 preparations each taken from 4 to 6 different animals). * *p* < 0.05 vs. CTRL, TTX, VIP 6–28 and VIP 6–28 + VIP (one-way ANOVA followed by Newman-Keuls post-test).

In the present experiments, ileal preparations (*n* = 32) showed spontaneous activity characterized by phasic rhythmic contractions (mean amplitude 4.903 ± 0.588 mN) (Figure 1A), which were unaffected by TTX (1 µmol/L, *n* = 4) (mean amplitude 5.100 ± 0.687 mN; *p* > 0.05) (Figure 1B,C), suggesting their myogenic origin.

The addition of VIP (100 nmol/L) to the bath medium (*n* = 12) caused a slight decay of the basal tension (mean amplitude 0.981 ± 0.098 mN) and a reduction in amplitude of the spontaneous contractions (mean amplitude 3.727 ± 0.294 mN) (Figure 1A–C). The effects of VIP on basal tension and on spontaneous motility were unaffected by 1 µmol/L TTX (*n* = 6; *p* > 0.05), indicating that they were directly exerted on the smooth muscle (Figure 1B,C). The addition of the VIP antagonist, VIP 6–28 (10 µmol/L), to the bath medium (*n* = 12) did not cause any effects per se on both basal tension and the amplitude of the spontaneous activity (*p* > 0.05), indicating the absence of a basal spontaneous VIP release from enteric neurons. VIP (100 nmol/L), added to the bath medium in the presence of 10 µmol/L VIP 6–28 (*n* = 6), no longer affected either the basal tension or the amplitude of the spontaneous contractions (Figure 1B,C).

The addition of GLP-2 (20 nmol/L) to the bath medium (*n* = 8), other than causing a slight decay of the basal tension (1.275 ± 0.196 mN), reduced the amplitude (mean amplitude 2.942 ± 0.490 mN) of ileal spontaneous contractions (*p* < 0.05) (Figure 1B,C).

To investigate the possible involvement of VIP in the inhibitory actions of the hormone, the latter was tested in the presence of the VIP antagonist. In the presence of 10 µmol/L VIP 6–28, GLP-2 (20 nmol/L, *n* = 6) still reduced the basal tension and the amplitude of spontaneous contractions (Figure 1B,C) to an extent comparable to GLP-2 alone (*p* > 0.05), indicating the lack of VIP involvement.

Further experiments were performed applying EFS to test whether VIP involvement in GLP-2 effects could emerge during neural activation.

#### 2.1.2. Neurally Induced Contractile Responses by EFS

**Figure 2:**
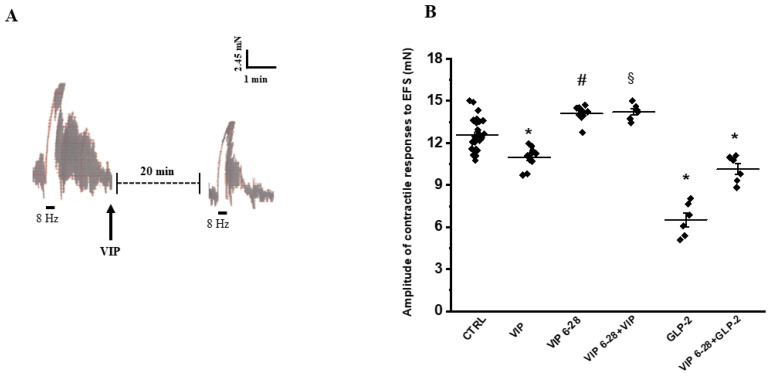
Effect of VIP on EFS-induced contractile responses in mouse ileal preparations. (**A**): Representative trace showing a contractile response evoked by EFS at 8 Hz stimulation frequency (left). Application of VIP (100 nmol/L) reduces the amplitude of the neurally mediated contraction (right). (**B**): Statistical analysis showing the mean amplitude of the EFS-evoked contractile responses under control conditions (CTRL), in the presence of VIP, VIP 6–28 or in their combination. GLP-2 significantly reduces the amplitude of EFS-evoked contractions compared to controls (CTRL). This inhibitory effect is attenuated when GLP-2 is applied in the presence of VIP 6–28. Values obtained following the addition of each substance to the organ bath were compared with their respective controls. As no significant differences were observed among control values, data were pooled and analyzed as a single control group (CTRL). Values are presented as mean ± SE (*n* = 6–10 preparations each taken from 4 to 8 different animals). * *p* < 0.05 vs. all other groups; # *p* < 0.05 vs. all other groups of values except VIP 6–28 + VIP; ^§^ *p* < 0.05 vs. all other groups of values except VIP 6–28 (one-way ANOVA followed by Newman-Keuls post-test).

In the present experiments, EFS at 8 Hz elicited a rapid phasic contraction (mean amplitude 12.544 ± 0.981 mN) superimposed on ongoing spontaneous activity (*n* = 28) (Figure 2A). The contractile response to EFS was abolished by either 1 µmol/L TTX (*n* = 4) or 1 µmol/L atropine (*n* = 4), indicating its nervous and cholinergic origin, respectively.

In the presence of VIP (100 nmol/L), a reduction in amplitude of the EFS-induced contractile responses (mean amplitude 10.787 ± 0.490 mN; *n* = 10) was observed (Figure 2A,B). In order to exclude that the reduction in amplitude of the EFS-induced contractile response by VIP might be due to a non-specific effect, the action of the VIP antagonist, VIP 6–28, was tested on the neurally induced contractile responses by EFS. Addition of VIP 6–28 (10 µmol/L) to the bath medium (*n* = 12) increased the amplitude of the neurally induced excitatory responses elicited by EFS (mean amplitude 14.119 ± 0.490 mN; *p* < 0.05) compared to controls (Figure 2B), thus indicating the removal of a weak inhibitory component caused by VIP release during 8 Hz stimulation frequency. In the presence of VIP 6–28, addition of VIP to the bath medium (*n* = 6) no longer affected the amplitude of the EFS-evoked contractile responses (mean amplitude 14.224 ± 0.588 mN) with respect to VIP 6–28 alone (*p* > 0.05) (Figure 2B).

The addition of GLP-2 at 20 nmol/L to the bath medium (*n* = 6) greatly reduced the amplitude of the EFS-induced contractile responses (mean amplitude 6.375 ± 1.079 mN; *p* < 0.05) (Figure 2B).

The effects of GLP-2 were tested on the EFS-induced contractile responses in the presence of VIP 6–28 (10 µmol/L). GLP-2 added to the bath medium in the presence of VIP 6–28 (*n* = 6) still depressed the amplitude of the EFS-induced excitatory responses (mean amplitude 9.807 ± 0.392 mN; *p* < 0.05) but to a lesser extent than with GLP-2 alone (Figure 2B), suggesting the involvement of VIP signaling in the effects of GLP-2 during EFS.

### 2.2. Immunohistochemical Results

#### Vasoactive Intestinal Peptide (VIP)-Immunoreactivity (IR) in the Ileal Muscle Coat

**Figure 3:**
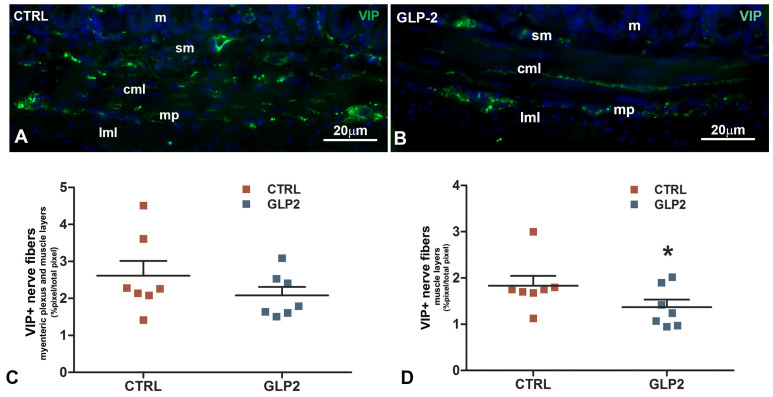
Vasoactive intestinal peptide (VIP) immunoreactivity (IR) in mouse ileal sections. In control (**A**) and in GLP-2 (**B**)-exposed sections, numerous VIP+ nerve fibers were observed at the myenteric plexus (mp) and in the thickness of the circular and longitudinal muscle layers (cml, lml). Quantitation of VIP nerve fibers in the muscle coat (**C**,**D**) showed a significant decrease (* *p* = 0.02; paired *t*-test) in GLP-2-exposed specimens only in the muscle layers (**D**). sm = submucosa; m = mucosa. Data are expressed as mean ± SE. (*n* = 7 mice). Bar = 20 µm.

VIP-IR was detected in numerous nerve fibers located at the myenteric plexus and in the thickness of the muscle layers, mostly in the circular one. The distribution was similar between controls and GLP-2-exposed specimens (Figure 3A,B). Quantitation of the VIP-IR in the entire muscle coat showed a decrease in GLP-2 preparations but the difference with CTRL did not reach the statistical significance (*p* > 0.05) (Figure 3C). Conversely, when VIP-positive nerve fibers were quantified separately, in the myenteric plexus and in the muscle layers, the decrease in GLP-2-treated specimens became significant in the latter *p* < 0.05 (Figure 3D).

## 3. Discussion

The results of the present study offer the first evidence that VIP signaling is involved in the inhibitory effects of GLP-2 on the neurally induced contractions in mouse ileal segments.

It is well established that during EFS, not only excitatory (mainly cholinergic) but also inhibitory (NANC) fibers are activated. The latter have been reported to release NO and, at the higher stimulation frequencies (≥8 Hz), also VIP in preparations from different regions of the gastrointestinal tract [35,36,37]. Interestingly, frequency-dependent release of VIP from myenteric neurons, with significantly enhanced VIP output at higher stimulation frequencies, has been specifically observed in the ileum of the guinea pig [38]. In the present experiments, the amplitude of the EFS-induced contractile responses at 8 Hz was increased by the VIP antagonist, suggesting the activation of the VIPergic inhibitory component at higher stimulation frequencies. Moreover, the observation that GLP-2 in the presence of the VIP antagonist depresses EFS-elicited contractile responses to a lesser extent compared to the hormone alone indicates the involvement of VIP signaling in the inhibitory effects of GLP-2 on neurally induced contractions. In this view, GLP-2R expression on enteric VIPergic neurons [39] and hormone-induced gastrointestinal smooth muscle relaxation in rodents through VIP release have been reported [31,32].

Notably, VIP added to the bath medium also causes a TTX-insensitive decay of the basal tension and a depression of the spontaneous contractions in unstimulated preparations, indicating its direct action on the smooth muscle. However, the lack of effects of VIP 6–28 per se on the spontaneous activity suggests that a basal spontaneous release of VIP from enteric neurons does not occur in ileal preparations. Moreover, the abolition of VIP effects by its antagonist not only confirms the efficacy of the antagonist itself but also rules out any non-specific effects of the peptide. The observation that the depressant actions of GLP-2 in unstimulated preparations were unaffected by the VIP antagonist suggests that the effects of the hormone on ileal spontaneous activity are not primarily mediated by VIP.

The immunohistochemical results give rise to a complex picture. VIP-positive nerve fibers were detected at the myenteric plexus and in the thickness of the smooth muscle layers, both in controls and GLP-2-exposed ileal segments. No positive neuronal bodies were appreciable at the myenteric plexus. Quantitation of the VIP labeling in the entire muscle coat showed a decrease in GLP-2-treated preparations that did not reach the significance. However, when the labeling was separately measured at the myenteric plexus and in the muscle layers, the VIP decrease became significant in the latter. These results are similar to those previously obtained in mouse gastric strips under comparable conditions [31].

However, in the present functional experiments, the role of VIP in the inhibitory effects of GLP-2 on the spontaneous activity was not observed. Specifically, in unstimulated preparations, the presence of the VIP antagonist did not alter the action of GLP-2. A possible explanation for this discrepancy between functional and immunohistochemical results may lie in the limited amount of VIP released by the hormone under these conditions, insufficient to impact spontaneous motility, despite the observed reduction in VIP labeling in GLP-2 exposed specimens. So, we hypothesize that this modest amount of VIP, released under basal conditions, is sufficient to cause a detectable reduction in VIP-IR, but not enough to alter motility in functional terms.

Conversely, the involvement of VIP in the inhibitory effects of GLP-2 became apparent during EFS. This may suggest that there is a recruitment of VIP by GLP-2 only when enteric VIPergic neurons are activated, as during EFS, a condition that cannot be reproduced for immunohistochemical investigations. In summary, the present findings are consistent with the notion that in the isolated ileum GLP-2 modulates the inhibitory neurotransmission through VIP recruitment, primarily under neuronal activation conditions. GLP-2-induced facilitation of VIP release from activated enteric neurons has also been reported in rodent gastric preparations [31,32]. Moreover, the modulatory effects of the hormone were already reported on the cholinergic and nitrergic neurotransmission too [23]. Thus, in addition to the ability of the hormone to decrease the cholinergic input to the smooth muscle and increase NO biosynthesis [23], the present findings indicate VIP signaling involvement during neuronal activation. These results suggest that GLP-2 may facilitate a coordinated modulation of multiple NANC inhibitory pathways, thereby optimizing intestinal relaxation under neuronal stimulation.

### Physiopathological Perspectives

From a physiological perspective, it could be speculated that this dual neuromodulatory action on NANC inhibitory neurotransmission, engaged by the hormone in the ileum, represents a synergic and reinforcing control system aimed at slowing intestinal transit, thereby improving nutrient absorption. Similarly, Amato and collaborators [32] proposed that the inhibitory actions of the hormone at the gastric level may be addressed to slow down the passage of food through the pylorus, leading to delayed gastric emptying, which is an advantageous effect for improving intestinal nutrient absorption. Therefore, the depressant effects of GLP-2 on ileal motility may be regarded as an additional redundant mechanism likely acting to further prolong the contact time between luminal contents and the intestinal mucosa, optimizing the efficiency of nutrient uptake, an effect which agrees with the role of the hormone in promoting intestinal absorption [8,9,10,11,12,13]. Moreover, this multiple control system on the enteric neurotransmission by GLP-2 might provide an efficient compensatory mechanism to overcome any possible deficit or dysregulation in neurotransmitter release, so preserving the efficacy of both intestinal motility and nutrient absorption and offering valuable insights into therapeutic strategies targeting gut dysmotility and absorption disorders [40,41]. Future studies using direct measurements of neurotransmitter release, receptor-specific tools, and in vivo models are essential to confirm these mechanisms and assess their clinical relevance.

Currently, GLP-2 analogs like teduglutide are approved for enhancing intestinal absorption in short bowel syndrome (SBS) [42], and other GLP-2 agonists are under development or investigation [43]. Recent reviews have further expanded on the potential therapeutic applications of GLP-2 analogs beyond SBS. These include possible benefits in functional gastrointestinal disorders involving dysmotility, malabsorption, or barrier dysfunction, although clinical validation remains limited at present [44]. Overall, GLP-2 analogs represent a growing class of gut-targeted hormonal therapies with established benefits in SBS and emerging roles in treating dysmotility and intestinal failure syndromes. In this context, GLP-2’s ability to modulate enteric neurotransmission by enhancing VIP and NO signaling while attenuating excitatory cholinergic activity is of particular interest, as it could help restore physiological motility patterns in patients with gut-brain axis imbalances [19]. Its capacity to modulate enteric neurotransmission opens new potential avenues for treating gastrointestinal dysmotility. In particular, targeting VIP-mediated pathways could help normalize excessive contractile activity and alleviate symptoms associated with dysregulated motility. Our present findings are consistent with a modulatory role of GLP-2 in ileal motility via VIPergic pathways, which primarily mediate smooth muscle relaxation and inhibition of contractility, as also evidenced by the sensitivity to the VIP antagonist, VIP 6–28. GLP-2 has been reported to promote VIP release primarily through activation of the GLP-2R expressed on enteric neurons [1,45]. Upon GLP-2 binding, GLP-2R initiates multiple intracellular signaling pathways including cAMP/PKA and PI3Kγ cascades, which have been implicated in VIP expression and neuronal plasticity [2,25]. These converging pathways contribute to several downstream effects of GLP-2, including improved intestinal barrier integrity, increased mucosal blood flow, and modulation of gut motility, all partially mediated by VIP release [46]. VIP acts mainly through two receptors, VPAC1 and VPAC2, both of which are targeted by VIP 6–28 [47]. Therefore, the present results do not allow us to differentiate the specific contributions of each receptor subtype, which may be relevant for the development of more targeted therapeutic strategies. Furthermore, it should also be emphasized that caution is warranted when translating findings from animal models to humans. Moreover, even if current scientific evidence suggests that GLP-2 does not exhibit major sex-related differences in its intestinotrophic effects across animal biological sexes [48], this aspect should also be carefully considered when developing therapeutic strategies. The lack of a direct assessment of VIP release, as well as the absence of use of selective VIP or GLP-2 receptor antagonists, represents a limitation that, if addressed, would further strengthen the present findings and would be essential to confirm the pathophysiological relevance of the observed effects. Therefore, further studies and clinical trials are needed to validate these therapeutic prospects and to understand the complex interactions between GLP-2, VIP, and other enteric neurotransmitters [49].

## 4. Materials and Methods

### 4.1. Animals and Ethical Approval

Experiments were conducted on 23 female C57BL/6J mice (ENVIGO, Udine, Italy), aged 8 to 12 weeks and weighing 21 ± 1.5 g. The animals were housed under standard laboratory conditions, including a 12 h light/dark cycle, a controlled temperature of 21 ± 1 °C, and ad libitum access to food and water. The experimental protocol was carried out in accordance with the European Communities Council Directive 2010/63/UE and the guidelines for the care and use of laboratory animals, as approved by the Animal Care Committee of the University of Florence (Italy), with authorization from the Italian Ministry of Health (authorization code 0DD9B.N.ZB6, approval date 15 October 2020). As previously described [23,50], and in agreement with the European Directive 2010/63/EU, animals were sacrificed by rapid cervical dislocation to minimize suffering. The abdomen was promptly opened, the distal ileum was carefully removed, and its luminal contents were gently rinsed with a physiological solution.

### 4.2. Functional Studies

Segments of the distal ileum, located within 30 mm of the ileocecal valve, were isolated. Transversely cut, two full-thickness preparations measuring 10 mm in length were dissected from each ileum and mounted in 5 mL double-jacketed organ baths filled with Krebs-Henseleit solution composed of 118 mM NaCl, 4.7 mM KCl, 1.2 mM MgSO_4_, 1.2 mM KH_2_PO_4_, 25 mM NaHCO_3_, 2.5 mM CaCl_2_, and 10 mM glucose, and continuously bubbled with a gas mixture of 95% O_2_ and 5% CO_2_ (pH 7.4). The solution temperature was kept at 37 °C (±0.5 °C) by circulating preheated water through the outer jacket of the organ bath using a thermostatically controlled circulator pump.

Each preparation was mounted along the axis of the longitudinal muscle layer, with one end secured to a platinum rod and the other attached to a force-displacement transducer (Grass model FT03, Quincy, MA, USA) via a silk thread, enabling continuous recording of isometric tension. The transducer was coupled to a polygraph system (Grass model 7K, Quincy, MA, USA). As previously described [23,50], the preparations were allowed to equilibrate for 1 h under an initial load of 14.71 mN. During this period, tissues were repeatedly and thoroughly washed with Krebs-Henseleit solution to prevent metabolite accumulation in the organ baths.

Electrical field stimulation (EFS) was delivered using two platinum wire rings (2 mm in diameter, spaced 5 mm apart) through which the preparation was threaded. Rectangular electrical pulses (80 V, 8 Hz, 0.5 ms duration, applied for 15 s) were generated by a Grass model S8 stimulator. The stimulation parameters were those previously shown to activate enteric neurons in ileal preparations [23].

#### 4.2.1. Drugs

The drugs utilized in this study included atropine (1 µmol/L), glucagon-like peptide-2 (GLP-2, 20 nmol/L), tetrodotoxin (TTX, 1 µmol/L), vasoactive intestinal peptide (VIP, 100 nmol/L), and the vasoactive intestinal peptide antagonist (VIP 6–28, 10 µmol/L).

All compounds were acquired from Sigma Chemical Company (St. Louis, MO, USA), except for GLP-2 and VIP 6–28, which were sourced from Tocris Bioscience (Bristol, UK).

Fresh solutions were prepared on the day experiments were conducted, except for TTX and VIP, which were stored as stock solutions at −20 °C.

Reported concentrations correspond to the final bath concentrations and align with those documented previously as effective in gastrointestinal preparations [23,24,31,32]. In particular, the doses of GLP-2 and VIP were selected based on the concentration-response curves [32,51], while the VIP 6–28 concentration was that previously reported to antagonize VIP-induced relaxation in the isolated mouse stomach [51].

#### 4.2.2. Experimental Protocol

In a first series of experiments, spontaneous contractile activity of ileal preparations was recorded both under control conditions and following application of the nerve terminal blocker tetrodotoxin (TTX).

In a second series of experiments, the influence of VIP on spontaneous motility was assessed, either alone or in combination with TTX or the VIP antagonist (VIP 6–28).

In the third series of experiments, the effects of GLP-2 on the spontaneous contractile activity were investigated alone or in the presence of the VIP antagonist (VIP 6–28).

In a fourth series of experiments, the responses to EFS were also recorded in the presence of TTX or atropine.

In a fifth series of experiments, the effects of VIP or the VIP antagonist (VIP 6–28) on the neurally induced contractile responses were evaluated also in their concomitant presence.

In a sixth series of experiments, the effects of GLP-2 added to the bath medium on the neurally induced contractile responses alone or in the presence of the VIP antagonist (VIP 6–28) were tested.

Based on our previous observation that, in the mouse gastric fundus strips [31], the response to a subsequent addition of GLP-2 at 20 nmol/L was restored only after a 15 min washout of the preparation, the same procedure was applied in the present experiments.

#### 4.2.3. Data Analysis and Statistical Tests

Contraction amplitudes, both spontaneous and nerve-induced, were quantified in absolute values (mN) at their peak response. Basal tension was evaluated as changes in the recording baseline and expressed as absolute values (mN). Control values were pooled across experiments only when no statistically significant differences were observed among them. Measurements taken after each drug administration were compared to their corresponding own control values. Statistical evaluation was performed using paired or unpaired Student’s *t* test or one-way ANOVA followed by the Newman-Keuls post-test for multiple comparisons, as in our previous studies [23,31]. Differences were considered statistically significant at *p* < 0.05. Results are expressed as means ± SE, and *n* represents the number of preparations studied. More than one measurement was performed on each preparation.

### 4.3. Morphological Studies

#### 4.3.1. Tissue Sampling

Seven animals were used; the ileum of each was removed and divided into two parts. One part of the ileum was used as a control, the adjacent part was exposed to GLP-2. At the end of the incubation, the ileal sections exposed for 30 min to GLP-2 (GLP-2) or those maintained in Krebs solution for the same period of time without the addition of the hormone (CTRL) were placed into a fixative solution, 4% paraformaldehyde solution (HT501320, Sigma-Aldrich, Merck, Darmstadt, Germany), overnight at 4 °C. The day after, the sections were dehydrated, cleared, and embedded in paraffin. Paraffin sections of 5 μm thickness were obtained with a rotary microtome (HistoCore MULTICUT, Leica, Buccinasco, Milan, Italy) and collected on slides.

#### 4.3.2. Immunohistochemistry

The sections, once deparaffinized and re-hydrated were treated for antigen retrieval for 20 min at 90–92 °C in Tris buffer (10 mM) with EDTA (1 mM, pH 9.0) then washed in 0.1 M phosphate-buffered saline (PBS, pH 7.4) and preincubated for 20 min in PBS plus 1.5% bovine serum albumin (BSA, AppliChem, Darmstadt, Germany) to lessen non-specific binding. Thereafter, the sections were exposed to VIP-H6 sc:25347 antibody (mouse monoclonal 1:100, Santa Cruz Biotech, Santa Cruz, CA, USA) overnight, in the same solution used for preincubation. The omission of the primary antibodies was used as negative control. The next day, the sections were incubated for 2 h at RT in the dark with appropriate fluorochrome-conjugated secondary antibodies (goat anti-mouse Alexa Fluor 488 A-10680 Invitrogen by Thermo Fisher Scientific, Hillsboro, OR, USA) diluted 1:333 in BSA 1.5% PBS. After washing, the sections were incubated for 10 min with Hoechst 33342, a nuclei marker (Enzo lifesciences, Farmingdale, NY, USA), washed in distilled water and mounted in an aqueous medium (Fluoromount-G, Invitrogen by Thermo Fisher Scientific, Carlsbad, CA, USA). The labeling was observed below an epifluorescence ZEISS Imager M.2 microscope (ZEISS, Oberkochen, Germany) at 488 and 465 nm excitation wavelengths for the green and the blue fluorescent labels, respectively, and the images were captured using a digital camera (Axiocam 305 mono, Zeiss, Oberkochen, Germany) and image acquisition software (Zeiss Zen 3.11).

#### 4.3.3. Quantitation and Statistical Analysis

The quantitation of the VIP-positive structures was performed on digitized images acquired with a 40x objective by using ImageJ software v1.53k (NIH, Bethesda, MD, USA) (two non-consecutive sections were used for each animal to quantify the labeling; 7 animals/group). The entire section was considered, and the ROI were traced along the thickness of the muscle coat to measure the total labeling; thereafter, several small ROI were designed at the level of the myenteric plexuses including the ganglia and the nerve strands. The same threshold was maintained for all sections, and the thresholding methods were standardized across samples. Pixels above the threshold were measured and expressed as positive pixels/total pixels. The analyses were performed blinded by two co-authors. GraphPad Prism 5.0 software (GraphPad, San Diego, CA, USA) was used for statistical analysis, and significant differences were assessed with the paired Student’s *t* test. A *p* < 0.05 was considered statistically significant.

## Data Availability

The original contributions presented in this study are included in the article. Further inquiries can be directed to the corresponding author.

## Abbreviations

The following abbreviations are used in this manuscript:

CTRL	control
EFS	electrical field stimulation
GLP-2	glucagon-like peptide-2
GLP-2R	glucagon-like peptide-2 receptor
NANC	non-adrenergic, non-cholinergic
L-NNA	L-NG-nitro arginine
NO	nitric oxide
TTX	tetrodotoxin
VIP	vasoactive intestinal peptide
SBS	short bowel syndrome
